# Molecular Method Is Essential to Identify Asymptomatic Malaria Reservoirs: A Successful Experience in the Malaria Elimination Program in Iran

**DOI:** 10.3390/diagnostics12123025

**Published:** 2022-12-02

**Authors:** Jebreil Shamseddin, Amin Ghanbarnejad, Abdoljabbar Zakeri, Farshid Abedi, Shaghayegh Khojasteh, Habibollah Turki

**Affiliations:** 1 Molecular Medicine Research Center, Hormozgan Health Institute, Hormozgan University of Medical Sciences, Bandar Abbas 7916613885, Iran; 2 Infectious and Tropical Diseases Research Center, Hormozgan Health Institute, Hormozgan University of Medical Sciences, Bandar Abbas 7919693116, Iran; 3 Social Determinants in Health Promotion Research Center, Hormozgan Health Institute, Hormozgan, University of Medical Sciences, Bandar Abbas 7919916753, Iran; 4 Infectious Diseases Research Center, Birjand University of Medical Sciences, Birjand 9717853577, Iran

**Keywords:** malaria, diagnostic, molecular techniques, low-density infection, case detection, elimination program

## Abstract

**Background:** The accurate diagnosis of malaria cases, especially asymptotic and low-parasitemia patients, using robust molecular methods (nested-PCR) have been emphasized. The goal of this study was to detect active cases of malaria in areas with a history of local malaria transmission focusing on the use of molecular tools to ensure that the malaria elimination program has been implemented successfully. **Methods:** In this cross-sectional study, 816 blood samples were taken from immigrants and local residents of malaria-endemic areas in Hormozgan province, Iran. In order to identify asymptomatic malaria parasite reservoirs, the samples were examined using microscopic, RDT, and nested-PCR techniques. **Results:** About twelve positive asymptomatic malaria cases were identified when the molecular method (nested-PCR) was used. The positivity rates among immigrants and local residents were 2.07% and 0.93%, respectively. No positive cases were detected using microscopic and RDT methods. **Conclusions:** The finding of the research emphasize that in addition to microscopy and RDTs methods, sensitive molecular tools as a standard and essential strategy are needed in the diagnosis and detection of asymptomatic parasite reservoir.

## 1. Introduction

Malaria is an infectious blood illness and the most important tropical disease in the world, which is taken into consideration in the programs of the World Health Organization. Malaria remains a serious health problem in tropical and subtropical regions of the world, despite a decline in cases over recent decades. According to a reported by the World Health Organization (WHO) in 2021, around 229 million cases of malaria were reported worldwide, with 409,000 deaths, and the local transmission of malaria continues in 77 countries [1,2]. Malaria has long been a serious health issue in Iran, resulting in significant economic and social losses [2]. Malaria’s impact on public health has prompted international organizations to focus on the disease, and malaria control, elimination and eradication programs have been extensively studied in recent years [3].

Following the strict implementation of malaria control programs and a significant reduction in positive cases, the malaria elimination program has been active in Iran with the technical support of the WHO since 2010 and is currently underway [2,3]. According to WHO criteria, Iran has entered the stage of the prevention of reintroduction, as local malaria transmission was not reported for two consecutive years in this country (2018, 2019). If no malaria local transmission is confirmed in the third year, the process of obtaining a malaria elimination certificate will begin [1,3,4]. Malaria imported cases, especially asymptomatic patients, as asymptomatic parasite reservoirs from neighboring malaria-endemic countries, are one of the most serious problems in Iran’s malaria elimination program. Therefore, it is also crucial to detect the disease in migrants.

Asymptomatic malaria reservoirs and low parasite cases are big challenges of malaria elimination programs [4]. As a result, every case (symptomatic and asymptomatic) must be detected, treated, and followed up on in the malaria elimination effort. In areas with a previous local malaria transmissions, accurate diagnosis using reliable laboratory methods is necessary to detect all types of infections, especially asymptomatic and low parasitemia [4].

Although microscope and RDT are routine diagnostic methods in the malaria endemic region, the use of molecularly sensitive techniques for the diagnosis of asymptomatic malaria is rapidly evolving [5].

The microscopic examination of stained peripheral blood smears is the standard method for the diagnosis of malaria. However, this specific, simple, and inexpensive method is not sensitive or effective in the diagnosis of low-parasitic cases, mixed infections, or asymptomatic parasitic reservoirs. In recent years, RDT method has been used to diagnose malaria, and research has shown that asymptomatic and low-parasitic cases cannot be easily identified using the RDT method. With respect to the above, the simultaneous use of molecular methods (Nested-PCR) has been emphasized as a very sensitive tool for malaria diagnosis, along with routine malaria diagnostic methods (Microscopy & RDT). Nested-PCR is a sensitive and accurate tool in the diagnosis of malaria, especially in low-parasitic and asymptomatic cases that cannot be detected by routine diagnostic methods [6,7].

Several studies in Iran and other countries have examined the efficiency of molecular detection in malaria control and elimination programs. Most researchers have suggested that the use of molecular techniques to detect asymptomatic parasitic reserves is essential in malaria elimination programs [5,6,7,8,9,10,11,12,13,14,15,16,17,18,19,20,21,22,23,24,25,26,27].

The goal of this study was to find asymptomatic malaria reservoirs in Hormozgan provincial areas having a history of local malaria transmission among indigenous populations and migrants, with a focus on the use of molecular tools to ensure that the malaria elimination program was implemented successfully. The finding of this study will aid in the identification of asymptomatic malaria reservoirs and monitoring strategies in majority cases in the area, as well as in all native malaria regions of Iran as a critical step toward malaria elimination across the country.

## 2. Materials and Methods

### 2.1. Study Area

**Figure 1:**
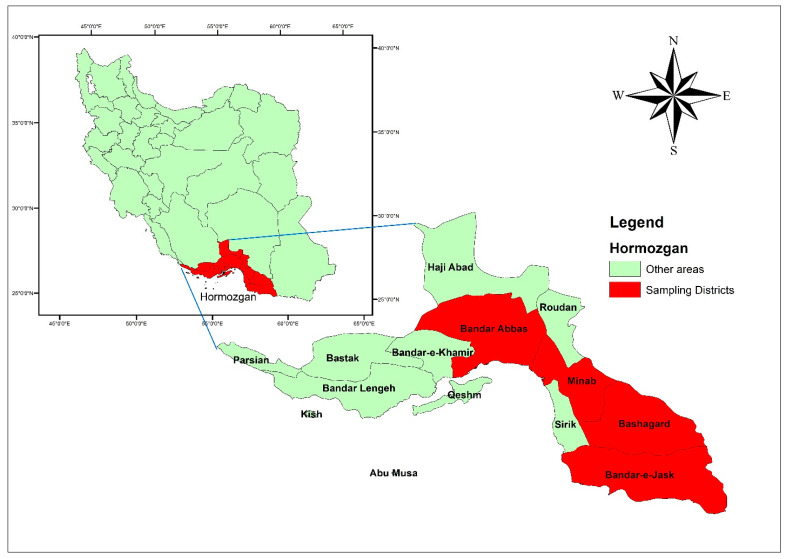
Map indicating the study areas; Hormozgan province.

This study was conducted in Hormozgan province as an area with a history of malaria transmission. According to the Portal of Hormozgan Governorate (http://www.Hormozgan.ir/, accessed on 15 May 2022), this is the southern province of Iran, which is located on the coast of the Persian Gulf and the Oman Sea with longitude and latitude of 27.4150° N and 56.7412° E, respectively. Its population is about 1,800,000 people and an area is about 68,000 square kilometers. This province is bordered by Kerman province from the north and northeast, Fars and Bushehr provinces from the northwest and west, and Sistan and Baluchistan province from the east. This province has 900 km of coastline with the Persian Gulf and the Sea of Oman. The capital of Hormozgan province is Bandar Abbas, and it has 13 counties, 40 districts, 45 cities, and 1277 villages (Figure 1).

### 2.2. Study Population

In this cross-sectional study, a total of 816 (57.6% male and 42.4% female) immigrants and local residents from malaria-endemic areas of Hormozgan were included in the study. The inclusion criteria for this study were as follows: people with a minimum age of 4 and a maximum of 65 years, not having clinical symptoms of malaria (chills, fever and sweating), not being treated with anti-malarial drugs, and not being pregnant. This study was approved by the ethics committee of the Vice Chancellor for Research and Technology of Hormozgan University of Medical Sciences (HUMS.REC.1396.40).

Sampling areas include Beshagard (*n* = 230), Minab (*n* = 200), Bandar Abbas (*n* = 330), and Jask (*n* = 56) in Hormozgan province.

Sample size calculation is based on this formula:

$$n=\frac{z^{2}pq}{d^{2}}=\frac{{\left(1.96\right)}^{2}\times \left(0.02\right)\times \left(0.98\right)}{{\left(0.01\right)}^{2}}\approx 753$$

Considering the asymptomatic malaria frequency of about 2%, confidence interval 95% and 0.01 difference between real and estimated positive percentage, the sample size was estimated to be 753. For preventing attrition in the study, 10% was added to the estimation, and finally 816 cases were investigated.

**Inclusion criteria:** healthy people living in areas with a history of malaria transmission, not receiving anti-malarial medication, non-pregnant women or not having clinical symptoms of malaria (chills, fever, sweating).

**Exclusion criteria:** The study was conducted cross-sectional, and individuals were selected after meeting the conditions for inclusion in the study, and no person was excluded from the study.

All participants filled out the consent form. Demographic characteristics (name, age, gender, occupation, immigration records and history of malaria) of people were recorded in a unique way. Participants were evaluated for the presence of clinical symptoms of malaria by physician.

### 2.3. Malaria Diagnosis Techniques:

About 2 mL of blood sample was taken from the participants for molecular analysis, and at the same time, thin and thick blood smears were obtained for microscopic malaria diagnosis. A rapid diagnostic test was also performed for all participants.

#### 2.3.1. Microscopic Analysis

A microscopic high-quality blood smears blood smear is the gold standard and a fundamental technique for laboratory detection of malaria which has suggested by WHO.

A sterile lancet was used to scrape the subjects’ fingertip. Next, the thin blood smears were prepared, while the thick blood smears were fixed with methanol. In order to find malaria parasites, the blood smear was then stained with Giemsa and examined under an immersion oil microscope at a magnification of ×1000 [28]. To confirm the accuracy of the microscopic data, blind professional microscopists reexamined the peripheral blood smears of all participants.

#### 2.3.2. Rapid Diagnostic Procedures (RDTs)

RDT and microscopy have been used in recent years in the effort to control and eliminate malaria. RDTs can identify parasite antigens such as *Plasmodium falciparum* histidine rich protein 2 (PfHRP-2), parasite lactate dehydrogenase (pLDH), aldolase, and glutamate dehydrogenase based on an immune-chromatographic approach (GDH) [29]. All study participants were examined using the RDT kit (Premier Medical Corporation Ltd., Mumbai, India). The RDT kits used in this study were capable of simultaneous detection of *Plasmodium falciparum* and *Plasmodium vivax*. Following the manufacturer’s instructions for the Malaria RDT kit, five microliters of blood drawn from a participant’s fingertip and added to a specific well in the RDTs kit. The RBCs were then hemolyzed and additional parasite antigen was released by adding three drops of lysing agent to the buffer well of the RDT cassette. The results of the test and control windows were evaluated based on the development of specific bands after ten minutes [29].

#### 2.3.3. Molecular Technique (Nested PCR)

The blood samples were transferred to the laboratory of the molecular medicine center of Hormozgan University of Medical Sciences under standard conditions for detection by PCR method. The sensitive molecular techniques yields more accurate result, according to the findings of earlier investigations [6,7,8,9,10,11,12,13,14,15,16,17,18,19]. The most sensitive method is nested-PCR, which detects Plasmodium parasite levels in blood at 2–5 parasites per microliter [30,31]. Consequently, in this study, a reliable and sensitive molecular approach was applied in addition to the routine methods (microscopy and RDT) of malaria diagnosing.

The molecular technique of nested PCR was utilized to identify malaria parasites in the samples in accordance with the methods described by Snounou et al. [32]. Genomic DNA Blood/Culture Cell Mini Kit from the Iranian company of Yekta Tajhiz Azma was used to isolate the parasite DNA from specimens. Two steps make up the Nested PCR process with two sets of primers. Firstly, the presence of Plasmodium is detected and secondly *P. falciparum* and *P. vivax* species is identified according to the selected primer. In the second stage of amplification, 2 µL of extracted DNA from each sample was added to additional reaction elements (nested PCR-1). The reaction is finished with a Plasmodium-specific primer (1200 bp). The reaction was conducted in a final volume of 50 µL, and the PCR was completed on a thermocycler with the necessary program. *P. vivax* (120 bp) and *P. falciparum* (205 bp) were identified using nested PCR-2, which uses the first-stage amplified product as the DNA template for the subsequent reaction. Each step was carried out 25–30 times for both procedures, with the annealing temperature set at 72 °C. The nested PCR-2 products were then examined using electrophoresis in the presence of suitable standard 100 bp molecular markers, and gel images were captured using digital imaging for the final report. Positive and negative controls along with PCR testing of the samples, were included in all reaction series. For negative controls, DNA was collected from the blood of healthy individuals who had never malaria and had no prior travel history to endemic regions. For the positive control preparation, parasite DNA was extracted from blood samples of people whose malaria had been verified by microscopic examination.

### 2.4. Data Analysis

The descriptive statistics used in the research including the report of frequency and percent for the variables. The data were analyzed using IBM SPSS version 25 software (IBM Corp., New York, NY, USA).

## 3. Results

The main characteristics of the study participants were shown in Table 1. Of the 816 studied cases, 57.6% were male and 42.4% were female. The people participating in the study were in four age groups, the highest frequency was in the 15–30 years group (48.8%) and the lowest frequency was in the over 45 years group (11.9%).

In the investigation using microscopic methods and RDT, no positive cases of malaria were reported. In order to ensure the accuracy of the results, the microscopic tests were performed by a double-blind skilled microscopist (Table 2 and Table 3).

In Table 4, the percentages of positive cases were reported according to gender and age groups. As can be seen in the table, the most prevalent age group for asymptomatic malaria was among 31 to 45 years (4.11%). The percent of positive cases were approximately the same across gender (male vs. female: 1.70% vs. 1.16%).

**Figure 2:**
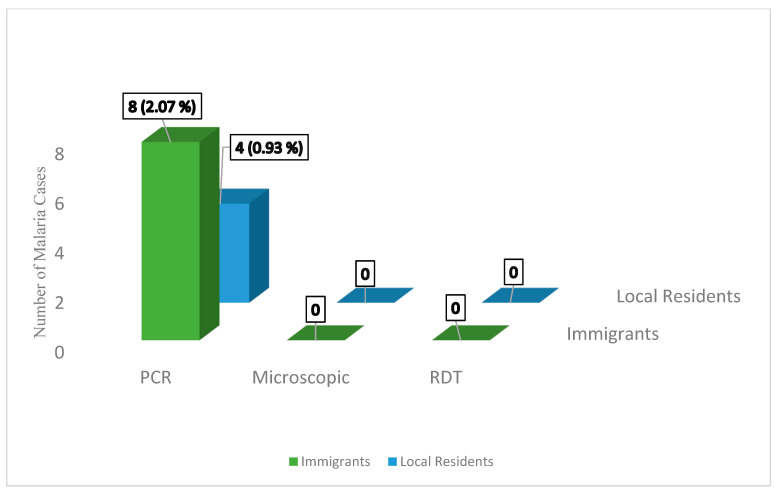
Detected positive cases by methods and nationality.

Using the molecular method (nested-PCR), 12 positive cases (10 *P. vivax* and 2 *P. falciparum*) were detected (Table 5). Most of the reported positive cases were from among the immigrants (Figure 2).

## 4. Discussion

The final goal of the malaria elimination program is to interrupt the local transmission of malaria. The malaria elimination program in Iran is in its final stages, and the most essential part of this project is to detect malaria cases in areas with a high risk of transmission. The WHO has underlined the importance of accurate malaria diagnosis and timely treatment as part of the malaria elimination mission. Toward the elimination of malaria, asymptomatic and low-parasitic infections are an important challenge. Asymptomatic malaria is an infection in which the patient has no symptoms. In malaria-endemic areas, continued exposure to Plasmodium parasites leads to asymptomatic carriers, which may contribute to continued transmission [15,16].

For successful malaria elimination, the use of comprehensive, rapid, robust and effective diagnostic technologies is necessary [33]. Microscopic and RDT are common procedures in the diagnosis of malaria which have some strengths and limits. PCR is the selected method for detecting low- parasite and asymptomatic cases [6,7].

Malaria elimination programs in different regions are an essential intervention strategy that targets the importance of predicting the malaria transmission situation in the study area and also the findings of molecular active cases [34,35].

This comprehensive study is aimed at identifying the challenges of the malaria elimination program with a focus on detecting asymptomatic malaria reservoirs in local residents and migrants using molecular approaches. In order to increase the accuracy of the results, microscopic, serological and molecular methods were used simultaneously. The microscopic method is standard method for malaria diagnosis, RDT is a supplementary approach, and nested-PCR with high sensitivity and specificity is used to detect low-parasite cases. Molecular technique revealed positive cases among immigrants and natives in Iran, despite all samples were found to be negative by microscopy and RDT methods. The findings of this study highlight the necessity of detecting asymptomatic reservoirs in malaria elimination programs using molecularly sensitive approaches, as well as the importance of malaria active case finding among local residents and migrants.

Dealing with the malaria elimination program would not be possible without considering the role of immigrants. As part of this study, the role of immigrants in the malaria elimination program in Iran was considered. Malaria is common among immigrants to Iran from Afghanistan and Pakistan. According to the results of this study, imported malaria is one of the biggest threats to the successful implementation of the malaria elimination program in Iran. The entry of asymptomatic malaria migrants into malaria-endemic areas of the country can re-establish the malaria transmission cycle due to the activity of malaria vectors at different times of the year and the availability of transmission conditions.

Several positive asymptomatic malaria cases reported were related to indigenous populations of malaria from endemic areas in Hormozgan, which is very important and could pose a crucial challenge and silent threat to the malaria elimination program in this region. Therefore, it is necessary to strengthen the malaria surveillance system to identify the malaria active foci in Hormozgan province, especially in the Minab and Beshagard regions, and implement enhanced interventions in line with the malaria elimination program.

The findings of this research are in line with previous Iranian investigations. In a study conducted in Hormozgan, Rashid et al. reported the presence of asymptomatic malaria cases in migrants by molecular methods [18]. In another study, the rate of asymptomatic malaria cases was reported to be 1.6 percent by microscopic approach in a study by Nateghpour et al. on Afghan refugees in Sistan Baluchestan province [26]. Furthermore, the Amirshekari research team used Nested-PCR to analyze asymptomatic malaria cases among Pakistani and Afghan migrants in Kerman province and found positive instances among both nationalities [36]. Findings from a study in Sri Lanka also highlight the destructive role of imported malaria in malaria elimination programs [37]. In another study in Hormozgan province, using molecular tools, Turki and colleagues identified three cases of asymptomatic malaria reservoirs in local residents of the Minab malaria endemic zone [15].

Many studies have emphasized that the application of several malaria detection methods simultaneously, with a focus on molecular methods to detect asymptomatic reservoirs, is critical in global malaria control and elimination efforts. This significant strategy is being employed in different parts of the world, and in all investigations a sensitive molecular technique has been used alongside conventional methods [5,6,7,8,9,10,11,12,13,14]. Quantitative molecular tools (qPCR) and nested-PCR are robust and sensitive methods in malaria diagnosis; in addition, some researchers showed that PET-PCR is effective in field conditions for molecular screening and examining a large number of samples [38,39,40].

The use of sensitive and robust malaria detection methods, appropriate sample sizes, and sampling of indigenous and immigrant populations in the area are the main advantages of the study. This research was conducted in a cross-sectional study and the lack of case follow-up is an important limitation of the study.

The setup of molecular detection in the study area is required according to WHO guidelines, in order to get a certificate of malaria elimination. As a result, it is recommended that a molecular laboratory be established in the centers nearest to malaria transmission regions.

## 5. Conclusions

Using molecular approaches and routine malaria testing, asymptomatic malaria reservoir were investigated. Asymptomatic parasite reservoirs, the majority among immigrants, were detected only by molecular methods.

The findings highlight the need to adopt molecular approaches in addition to microscopy and RDTs as standard strategies for the diagnosis and detection of asymptomatic parasite reservoir.

This study also shows that the imported cases of asymptomatic malaria in Hormozgan, together with the suitable climatic conditions for malaria transmission, are one of the significant challenges for the malaria elimination program. In order to successfully implement the malaria elimination program, it is suggested to set up molecular methods with more applicability in the field such as PET-PCR and LAMP in areas with local transmission of malaria.

## Figures and Tables

**Table 1:** Demographic characteristics of the participants in an asymptomatic malaria study.

Variable	Category	No. (*n* = 816)	Percent
Age Group	<15 years	175	21.4%
	15–30 years	398	48.8%
	30–45 years	146	17.9%
	>45 years	97	11.9%
Gender	Female	346	42.4%
	Male	470	57.6%
Location	Bandar Abbas	330	40.4%
	Bashagard	230	6.9%
	Minab	200	24.5%
	Jask	56	28.2%
Residency	Local residents	430	52.7%
	Immigrants	386	47.3%

**Table 2:** Positive cases according to sampling location among local residents in an asymptomatic malaria study.

Sampling Location	No. of Positive Cases by Methods			Total Surveyed Cases	Percent of Positivity with PCR
	PCR	Microscopic	RDT		
Bashagard	1	0	0	230	0.43%
Minab	3	0	0	200	1.5%
Total	4	0	0	430	0.93%

**Table 3:** Positive cases according to sampling location among immigrants in an asymptomatic malaria study.

Sampling Location	No. of Positive Cases by Methods			Total Surveyed Cases	Percent of Positivity with PCR
	PCR	Microscopic	RDT		
Bandar Abbas	5	0	0	330	1.52%
Jask	3	0	0	56	5.36%
Total	8	0	0	386	2.07%

**Table 4:** Positive malaria cases detected by PCR method according to gender and age group in an asymptomatic malaria study.

Variable	Category	Total Number	No. of Positive Cases	Percent of Positive Cases
Age Group	<15 years	175	1	0.57%
	15–30 years	398	5	1.26%
	31–45 years	146	6	4.11%
	>45 years	97	0	0%
Gender	Female	346	4	1.16%
	Male	470	8	1.70%

**Table 5:** Positive malaria cases detected by PCR according to parasite species, location, and residency in an asymptomatic malaria study.

Location	Residency	No. of Positive Cases		Percent of Positive Cases	
		P.v	P.f	P.v	P.f
Bandar Abbas	Immigrants	4	1	1.21%	0.3%
Jask	Immigrants	2	1	3.57%	1.79%
Bashagard	Local	1	0	0.43%	0%
Minab	Local	3	0	1.5%	0%
Sampling areas	Immigrants	6	2	2.07%	0.52%
	Local	4	0	0.93%	0%
	Total	10	2	1.23%	0.25%

## Data Availability

The general data obtained from the study and the results of the analyzes performed on these data will be provided by the corresponding author whenever needed.
